# Characterization of long‐term survivors with liver metastases from uveal melanoma diagnosed between 2005 and 2021

**DOI:** 10.1002/ijc.70246

**Published:** 2025-11-11

**Authors:** Jona Laukhuf, Lisa Wiens, Gerd Grözinger, Helmut Dittmann, Karolin Thiel, Ulrike Leiter, Teresa Amaral, Lena Nanz, Lukas Flatz, Markus Reitmajer, Andrea Forschner

**Affiliations:** ^1^ Department of Dermatology University Hospital Tuebingen Tuebingen Germany; ^2^ Department of Diagnostic and Interventional Radiology University Hospital Tuebingen Tuebingen Germany; ^3^ Center of Radiology, Minimally Invasive Therapies and Nuclear Medicine, SLK‐Kliniken Heilbronn Academic Hospital of Ruprecht‐Karls‐University Heidelberg Germany; ^4^ Department of Nuclear Medicine and Clinical Molecular Imaging Eberhard Karls University of Tuebingen Tuebingen Germany; ^5^ Department of General, Visceral and Thorax Surgery Oberschwabenklinik Ravensburg Germany

**Keywords:** cancer survivors, liver metastases, long‐term survivors, metastatic uveal melanoma, survivorship

## Abstract

Patients with metastatic uveal melanoma (UM) have a poor prognosis. While long‐term survival data in cutaneous melanoma (CM) are promising, such data are lacking in UM and long‐term survivors are rare. In cases of metastases, the liver is affected in 90% of cases and is the main determinant of life expectancy. Immune checkpoint inhibitors (ICI) are less effective in UM than in CM. Therefore, liver‐directed therapies are of high relevance. We evaluated a large cohort of UM patients (*n* = 167) who had developed liver metastases between 2005 and 2021. We focused on patients who survived 3 years or more from the initial diagnosis of liver metastases and precisely characterized this cohort with regard to systemic therapies (ST) and liver‐specific procedures applied. The last follow‐up (FU) date was October 31, 2024. We identified 33 long‐term survivors, representing 20% of the total cohort. Most patients had additional extrahepatic metastases (23/33; 70%), while 10 patients had liver metastases only. First‐line treatment in the metastatic setting consisted of liver‐directed therapies in most of the cases (27/33; 82%). 90% of the patients had received at least one liver‐specific procedure at any time point during FU and 85% had received ICI at any time point. Response evaluation revealed a high percentage of patients with disease control (DC) after the first ST (17/27; 63%) or first liver‐specific therapy (27/29; 93%), respectively. Notably, all patients with chemosaturation as their first liver‐specific procedure achieved DC. Further data are needed on the combination of liver‐directed therapy and ST, such as ICI or Tebentafusp.

## What's New?

Liver metastasis is an indicator of life expectancy for patients with advanced uveal melanoma (UM). Data on anticipated survival time following the diagnosis of liver metastasis, however, is lacking. The present study analyzed long‐term survival among UM patients who lived three or more years after liver metastases were diagnosed. Although outcomes for metastatic UM were generally poor, one‐fifth of patients survived three or more years. Most patients developed extrahepatic metastases in addition to liver metastases, and the majority showed disease control after the first liver‐specific therapy. Further investigation of survival among UM patients following systemic versus liver‐directed treatment is warranted.

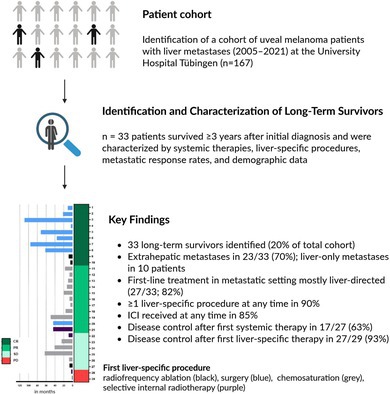

**Abbreviations**
CM: cutaneous melanoma
CMMR: Central Malignant Melanoma Registry
CR: complete response
DC: disease control
ECOG: Eastern Cooperative Oncology Group
FU: follow‐up
ICI: immune checkpoint inhibitors
IQR: interquartile range
LT: liver‐specific therapies
MSS: melanoma‐specific survival
ORR: objective response rate
OS: overall survival
PD: progressive disease
PFS: progression‐free survival
PHP: percutaneous hepatic perfusion
PR: partial response
RECIST: response evaluation criteria in solid tumors
SD: stable disease
ST: systemic therapies
TT: targeted therapies
UM: uveal melanoma

## 1 INTRODUCTION

Long‐term survival in stage IV cutaneous melanoma (CM) has become increasingly achievable with the approval of targeted therapies (TT) and immune checkpoint inhibitors (ICI).^1^, ^2^, ^3^ For patients treated with first‐line combination ICI using ipilimumab and nivolumab, the 10‐year melanoma‐specific survival (MSS) exceeds 50%.^3^, ^4^ However, metastatic uveal melanoma (UM) still exhibits poor survival.^5^, ^6^, ^7^ Three‐year follow‐up (FU) data from Tebentafusp, a T‐cell receptor‐bispecific molecule targeting glycoprotein 100 and CD3, that had been approved for metastatic UM, reports a median overall survival (OS) of 21.6 months in the Tebentafusp group compared to 16.9 months in the control group (investigator's choice). The estimated 3‐year survival rate was 27% in the Tebentafusp group and 18% in the control group.^8^ A retrospective analysis of 89 patients with metastatic UM found a similar median OS of 21.8 months (95% confidence interval (CI), 16.6–31.3). Additionally, this study identified the development of extrahepatic‐only metastases, an Eastern Cooperative Oncology Group (ECOG) performance status of 0, prior ICI, and female sex as factors associated with more than a two‐fold reduction in mortality risk.^9^

Systemic therapy in UM still has its limits, which makes liver‐directed procedures such as percutaneous hepatic perfusion (PHP) (also called chemosaturation) highly relevant. For the PHP procedure high‐dose chemotherapy (Melphalan) is directly applied to the hepatic arteries while the venous outflow of the liver is vacuumed up and filtered by an extracorporeal filter system to avoid systemic effects of Melphalan. A multicentric Phase III trial including patients with unresectable metastasized UM receiving Melphalan‐PHP showed an objective response rate (ORR) of 36.3%, a median progression‐free survival (PFS) of 9 months and a median OS of 20.5 months.^10^ This highlights the promising outcomes of PHP. Another retrospective study showed that UM patients with liver metastases receiving first‐line Melphalan‐PHP treatment showed significantly higher hepatic PFS (median: 17.6 months) and overall PFS (median: 15.4 months) compared to a group of patients receiving immunotherapy or other liver‐directed therapies. Similar outcomes were shown in patients receiving PHP as a second‐line treatment.^11^

There is a general trend that liver‐directed therapy is associated with higher survival rates: A retrospective analysis showed a correlation between higher survival and first‐line liver‐directed therapies than first‐line systemic therapies (ST). Furthermore, the median MSS was higher in patients receiving liver‐directed therapy (MSS: 28 months) than in those patients receiving systemic therapy as a first therapy (MSS: 10 months).^4^

In this study, we present data on UM long‐term survivors, defined as patients who survived three or more years following the initial diagnosis of liver metastases. We provide a comprehensive characterization of this cohort, with a particular focus on ST and liver‐directed treatment strategies.

## 2 METHODS

### 2.1 Study design

The study was designed as a retrospective analysis of a large cohort of patients diagnosed with metastatic UM who developed liver metastases between 2005 and 2021 and had been treated at the University Hospital of Tuebingen. The patients were identified using the Central Malignant Melanoma Registry (CMMR).

We collected information from the medical records on the type of systemic therapy and liver‐specific therapy received as a treatment of the metastases from UM. The last FU date was October 31, 2024.

Liver‐specific therapies (LT) included radiofrequency ablation, surgery, stereotactic radiosurgery, chemosaturation, radioembolization, and transarterial chemoembolization.

ST were differentiated by immune ICI, chemotherapy and other ST.

Response assessment was based on the medical records including the information provided by the interdisciplinary tumor boards and grouped according to the revised response evaluation criteria in solid tumors (RECIST) guidelines (version 1.1)^12^ with complete response (CR), partial response (PR), stable disease (SD), or progressive disease (PD) as possible outcomes. SD, PR and CR are all defined as disease control (DC).

The median OS was defined as the time between the start of the first liver‐specific treatment or first systemic therapy and death or last contact date if the patient was alive. Progression‐free survival (PFS) was defined as the time between the start of treatment and progression and death or last contact date if the patient was alive.

### 2.2 Statistical analysis

Descriptive statistical analysis was performed using IBM SPSS Statistics 28.0.0.0 (IBM, New York, USA). Figures were designed using GraphPad PRISM 9.5.0 (Dotmatics, Boston, USA). Categorical variables are given as absolute frequencies and percentages, while quantitative variables are shown as medians with interquartile ranges (IQR) (Table 1).

**TABLE 1:** Baseline patient characteristics total cohort.

	*N*	%
Female	21	63.6
Male	12	36.4
Months between initial diagnosis and first liver metastasis		
Median [range]	36	[2–338]
Age at the time of first liver metastasis		
Median [range]	62	[41–85]
Age at the time of first diagnosis of UM		
Median [range]	56	[35–82]
Months between first liver metastasis and last contact		
Median [range]	55	[36–126]
Presence of liver metastases	33	100.0
Only liver metastases	10	30.3
Still alive at the time of evaluation	15	45.5
Still receiving systemic therapy at the time of evaluation	2	6.1
ICI at any time point		
Yes	28	84.8
No	5	15.2
Liver‐specific therapy at any time point		
Yes (chemosaturation included)	30	90.9
No liver‐specific local therapy	3	9.1
Chemosaturation at any time point		
Yes (chemosaturation performed)	17	51.5
No (chemosaturation not performed)	16	48.5
≥1 systemic therapy	28	84.5
≥2 systemic therapies	19	57.6
≥3 systemic therapies	9	27.3

*Note*: Following abbreviations are used: no data available (n/d), central nervous system (CNS).

Abbreviations: ICI, immune checkpoint inhibitors; UM, uveal melanoma.

## 3 RESULTS

### 3.1 Patient characteristics

One hundred and sixty‐seven patients with UM and liver metastases diagnosed at the University Hospital of Tuebingen between 2005 and 2021 were identified from the CMMR. Twenty percent of these patients (*n* = 33) had an OS of at least 3 years from the initial diagnosis of liver metastases. These patients represent the entire cohort of long‐term survivors. Of the patients in this cohort, 63.6% were female and 36.4% were male.

The median time period between the initial diagnosis of UM and first detection of liver metastasis was 36 months (IQR = 83.5 months). The median age of the patients at the time of liver metastasis was 62 years (IQR = 17.5 years) (Table 1).

### 3.2 Applied systemic therapies

**FIGURE 1:**
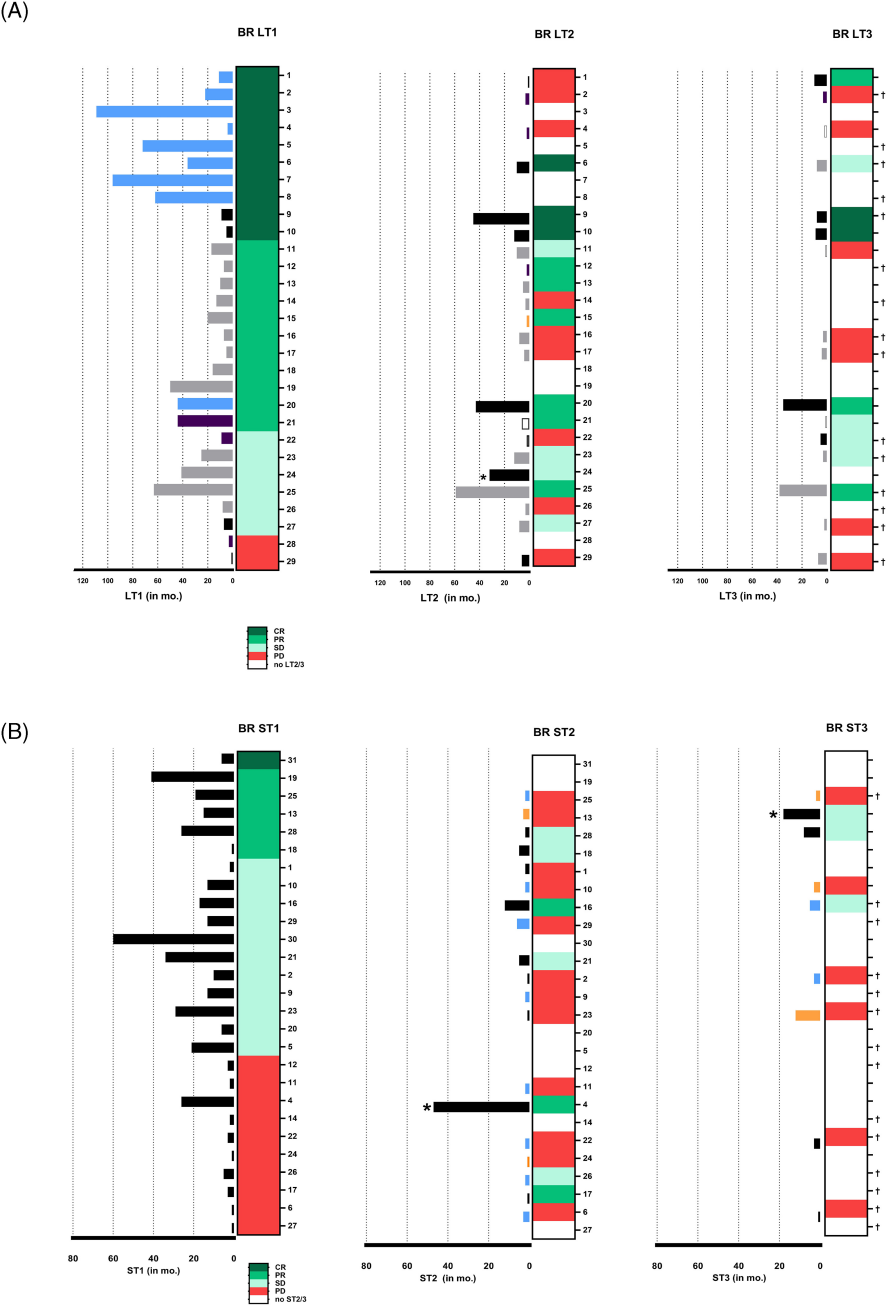
(A) Best response and time to progression of first three liver‐specific therapies (LT). Each bar represents the time (in months) between a LT and the time to progression after that therapy for each individual patient whose number is noted to the right of the response column. The following color code is used for the LT: radiofrequency ablation (black), surgery (blue), stereotactic radiosurgery (orange), chemosaturation (gray), selective internal radiotherapy (purple) and transarterial chemoembolization (white with black border). One patient was excluded due to missing data on the best response of therapy. Three patients did not receive any LT and are therefore not shown in the figure. Deceased patients are indicated by †. One patient who did not experienced disease progression after the most recent liver therapy is marked with (*). The length of the bar for this patient reflects the period between the date of liver therapy and the last documented date of contact. The vertical column shows the best response with each LT. Dark green: Complete remission (CR) or no evidence of disease, medium green: partial remission (PR), light green: stable disease (SD), red: progressive disease (PD). (B) Best response and duration of first three systemic therapies (ST). Each bar shows the duration of ST (in months). The following color code is used: Immune checkpoint inhibition (black), chemotherapy (blue) and other therapies (orange). One patient was excluded due to missing data on the best response and duration of therapy. Five patients did not receive any ST. Deceased patients are indicated by †. Patients still receiving ST at the time of evaluation are marked with (*). The vertical column shows the best response with each ST. Dark green: CR, medium green: PR, light green: SD, red: PD.

Most of the long‐term survivors (85%, 28/33) had received ICI therapy at any time point. The majority of these patients (63%, 17/27 [unknown response for one patient]) showed DC on the first systemic therapy applied, which was ICI in all these patients (28/28, 100%). ICI therapy consisted of ipilimumab monotherapy, ipilimumab and nivolumab combined, pembrolizumab or nivolumab monotherapy. The median PFS in patients receiving ICI as first systemic therapy was 5.55 months and the OS was 35.99 months. The greater part of patients (17/28, 61%) had combined ipilimumab/nivolumab therapy. About one‐third (27%) of patients received at least three ST in total (Figure 1, Table S2). Regarding the patients who did not receive ICI therapy at any time (15%, 5/33), four patients received only liver‐directed therapy, and one patient received a study therapy. It is of interest that in these patients (*n* = 5), long‐term survival was seen despite not receiving ICI therapy at any time point.

### 3.3 Liver‐specific therapies

Response evaluation revealed a high percentage of patients with DC at the first liver‐specific therapy (27/29; 93%).

First‐line treatment after the diagnosis of liver metastases consisted in most of the cases of LT (27/33; 82%). Most of the patients (90%) received at least one liver‐specific procedure at any time point until the last date of FU. 49% of the patients received at least three LT in total. 89% (8/9) of the patients receiving surgery as a first liver‐specific therapy showed a CR (Figure 1A). A detailed description of the LT received can be found in Table S1.

### 3.4 Chemosaturation therapy

Fifty‐two percent of the patients received chemosaturation at any time point. The median OS of those patients was 37.35 months, with the median PFS 10.28 months. The majority of patients (16/17; 94.12%) receiving chemosaturation also received ICI therapy, while 75% (12/16) of the patients that did not receive chemosaturation, received ICI. All patients (13/13, 100%) that received chemosaturation as the first liver‐specific therapy had DC. The best response in nine of those patients (9/13, 69%) was PR, while three of the patients had SD.

## 4 DISCUSSION

Metastatic UM remains a clinical challenge with limited treatment options and poor prognosis.^4^, ^5^ When complete resection of metastases is feasible in patients with UM, surgery is typically the preferred approach and is associated with the most favorable outcomes as first liver‐directed therapy.^4^, ^13^ If liver metastases cannot be removed, chemosaturation is an effective treatment approach. It is remarkable that all patients in our cohort with chemosaturation as a first liver‐specific therapy achieved DC. Regarding the ST we have found that most patients had DC on ICI therapy. It has to be considered, that 68% (19/28) of these patients had additional extrahepatic metastases.

Since most patients in our specific cohort had extrahepatic metastases and a relatively large number of patients achieved DC with ICI, a potential link between the presence of extrahepatic metastases and response to ICI therapy could be assumed in our cohort. To further validate this observation further prospective studies are needed.

In addition, there are several publications reporting on worse outcomes in patients with liver metastases in patients with melanoma and ICI.^14^, ^15^

The reasons for the reduced efficacy of ICI in liver metastases are not completely understood. In general, the liver is known to be able to induce immune tolerance and thus suppress immune activation.^16^ One explanation could be the reduced number of CD3+ T cells in liver metastases compared to other metastases. Therefore, the microenvironment of the liver might directly contribute to an inhibition of effective antitumor effects of ICI.^17^

For these reasons, the combination of ICI (for extrahepatic tumor control) and PHP (for hepatic tumor control) seems to be reasonable.

A phase Ib trial combining ICI therapy with Melphalan‐PHP revealed that Melphalan‐PHP combined with 1 mg/kg ipilimumab and 3 mg/kg nivolumab seems to be a safe treatment approach. The median PFS in these patients was 29.1 months, the ORR 85.7% and the DC rate 100%.^18^ Compared to the results of PHP or ICI alone, the results of a combinatory treatment approach are promising. The main idea of the study was based on the hypothesis that hepatic metastases can be controlled with PHP while extrahepatic metastases need to be controlled with systemic therapy such as ICI therapy.

Many patients in our cohort of long‐term survivors received both, ICI and PHP therapy (*n* = 15). This also indicates that the combined approach appears to be associated with a better outcome. Twelve patients in our cohort only had PHP, without additional ICI as they had only hepatic metastases. The high chance of effective DC with PHP therapy is underlined by the studies of Ghali et al. and Zager et al. that highlight excellent response rates and long PFS in patients with metastasized UM and PHP therapy.^10^, ^11^ In the case of additional extrahepatic metastases, additional systemic treatment approaches such as ICI or Tebentafusp are warranted.

Current data^8^ revealed promising data on OS in UM patients receiving Tebentafusp. Therefore, studies on combined treatment approaches with PHP and Tebentafusp are needed in the future.

## 5 LIMITATIONS OF THIS STUDY

The main limitations of this study are the small cohort size (*n* = 33) and the retrospective, single‐center study design at a university hospital. Because of the retrospective design, a lead‐time bias cannot be ruled out, as imaging of the entire cohort prior to distant metastasis was not performed at uniform time points. The observation of the retrospective cohort started with the first distant metastasis. Nevertheless, our study contains relevant findings that have not yet been reported in such detail. It is an important finding that even in the metastatic setting of UM patients, long‐term survival can be achieved in individual cases.

## AUTHOR CONTRIBUTIONS

**Jona Laukhuf:** Conceptualization; visualization; data curation; methodology; software; investigation; validation; writing – original draft. **Lisa Wiens:** Writing – review and editing; data curation; software. **Gerd Grözinger:** Writing – review and editing. **Helmut Dittmann:** Writing – review and editing. **Karolin Thiel:** Writing – review and editing. **Ulrike Leiter:** Writing – review and editing; data curation; software. **Teresa Amaral:** Writing – review and editing. **Lena Nanz:** Writing – review and editing; data curation; software. **Lukas Flatz:** Writing – review and editing. **Markus Reitmajer:** Writing – original draft; writing – review and editing; conceptualization; visualization; data curation; methodology; software; validation; investigation. **Andrea Forschner:** Writing – original draft; writing – review and editing; conceptualization; data curation; methodology; validation; software; supervision; project administration.

## FUNDING INFORMATION

Markus Reitmajer received funding as part of the Junior Clinician Scientists Program of the University of Tuebingen (application no. 523‐0‐0). Otherwise, this research has not received any specific grants from public, commercial, or non‐profit organizations.

## CONFLICT OF INTEREST STATEMENT

Jona Laukhuf received financial support for travel and congress participation from Delcath. This support was unrelated to the study design, data analysis, or manuscript preparation. Lisa Wiens declares no conflict of interest. Gerd Grözinger declares no conflict of interest. Helmut Dittmann received honoraria for advisory or and speaker's fees from Novartis, Bayer, GE Healthcare, IPSEN and Recordati Rare Diseases not related to the submitted work. Karolin Thiel declares no conflict of interest. Ulrike Leiter received travel support and speaker's fees and advisor's honoraria by Novartis, MSD, Pierre Fabre, Regeneron, Sanofi, Sun Pharma, Almirall Hermal and research funding from MSD, outside the submitted work. Teresa Amaral reports personal fees for advisory board membership from Delcath and Philogen; personal fees as an invited speaker from Bristol Myers Squibb (BMS), Medscape, Neracare, Novartis, and Pierre Fabre; personal fees for a writing engagement from CeCaVa and Medtrix; institutional fees as local principal investigator (PI) from Agenus Inc., AstraZeneca, BioNTech, BMS, HUYA Bioscience, Immunocore, IO Biotech, MSD, Pfizer, Philogen, Regeneron, Roche and University Hospital Essen; institutional fees as coordinating PI from Unicancer; institutional research grants from iFIT and Novartis; institutional funding from MNI—Naturwissenschaftliches und Medizinisches Institut, Neracare, Novartis, Pascoe, Sanofi and Skyline‐Dx; non‐remunerated membership of the American Society of Clinical Oncology (ASCO) and the Portuguese Society for Medical Oncology; a role as clinical expert in the area of medical oncology for Infarmed, and a role as an expert for SGA‐Oncology at EMA. Lena Nanz declares no conflict of interest. Lukas Flatz received grants from Hookipa Pharma, Swiss Cancer League, German Research Foundation, Immunophotonics, Mundipharma. Lukas Flatz received consulting fees from Philogen and support for attending meetings or travel from Philogen, Hookipa Pharma. Lukas Flatz participates on the board for the University of Basel (TIL trial, unpaid) and is the founder of Hookipa Pharma, Schmelzberg, Humion, and Abtherix—all outside the submitted work. Markus Reitmajer received funding as part of the Clinician Scientist Program of the University of Tuebingen (application no. 523‐0‐0) and travel support from Almirall Hermal, Galderma, and Pierre‐Fabre, outside the submitted work. Andrea Forschner received travel support and/or speaker's fees and/or advisor's honoraria from Novartis, BMS, MSD, Pierre Fabre, Delcath, Immunocore and research funding from Stiftung Immunonkologie BMS and Hiege Stiftung gegen Hautkrebs, all outside the submitted work.

## ETHICS STATEMENT

All patients included in this registry provided written informed consent for documentation of their clinical data in the central melanoma registry for research purposes and publications. Approval from the Ethical Committee of the University Tuebingen was obtained (reference number 806/2022BO2) and followed the general recommendations outlined in the Declaration of Helsinki.

## Supporting information


**Data S1.** Supporting Information.

## Data Availability

The data that support the findings of this study are available from the corresponding author upon reasonable request.
