# Supplementary material for: Characterization of long‐term survivors with liver metastases from uveal melanoma diagnosed between 2005 and 2021
Source: Int J Cancer. 2025 Nov 11;158(8):2179–85. doi: 10.1002/ijc.70246 (PMC12922641; doi:10.1002/ijc.70246)
Supplement: Supplementary file 1 — Data S1. Supporting Information. [file IJC-158-2179-s001.pdf]

Supplementary material

Characterization of long-term survivors with liver metastases from uveal melanoma diagnosed between 2005 and 2021

Jona Laukhuf, Lisa Wiens, Gerd Grözinger, Helmut Dittmann, Karolin Thiel, Ulrike Leiter, Teresa Amaral, Lena Nanz, Lukas Flatz, Markus Reitmajer*, Andrea Forschner*

Supplementary Table 1: Applied liver-specific therapies 2
Supplementary Table 2: Applied systemic therapies 3

**Liver-specific therapy**	**N**	**%**
**Received at least one course of liver specific therapy**	**16/33**	**48.4**
– Surgery as first liver-specific therapy	5/16	31.3
– Chemosaturation as first liver-specific therapy	5/16	31.3
– RFA as first liver-specific therapy	4/16	25.0
– SIRT as first liver-specific therapy	2/16	12.5
**Received at least two courses of liver specific therapy**	**16/33**	**48.4**
– RFA as second liver-specific therapy	6/16	37.5
– Chemosaturation as second liver-specific therapy	6/16	37.5
– SIRT as second liver-specific therapy	2/16	12.5
– TACE as second liver-specific therapy	2/16	12.5
**Received at least three courses of liver specific therapy**	**16/33**	**48.4**
– Chemosaturation as third liver-specific therapy	8/16	50.0
– RFA as third liver-specific therapy	4/16	25.0
– TACE as third liver-specific therapy	3/16	18.8
– SIRT as third liver-specific therapy	1/16	6.3

Supplementary Table 1: Applied liver-specific therapies

Following abbreviations are used: Radiofrequency Ablation (RFA), Transarterial Chemoembolization (TACE), Selective Internal Radiation Therapy (SIRT)

Systemic therapy	N	%
**Received at least one course of systemic therapy**	**28/33**	**84.8**
– ICI as first systemic therapy	28/28	100
○ *Ipilimumab monotherapy*	*3/28*	*9.1*
○ *Nivolumab monotherapy*	*5/28*	*15.2*
○ *Ipilimumab/Nivolumab*	*17/28*	*51.5*
○ *Pembrolizumab*	*3/28*	*9.1*
**Received at least two courses of systemic therapy**	**19/33**	**57.6**
– ICI as second systemic therapy	9/19	47.4
○ *Ipilimumab monotherapy*	*1/9*	*11.1*
○ *Nivolumab monotherapy*	*2/9*	*22.2*
○ *Ipilimumab/Nivolumab*	*6/9*	*66.7*
– Chemotherapy as second systemic therapy	8/19	42.1
– Other therapies as second systemic therapy	2/19	10.5
○ *Whole body hyperthermia combined with misteltoe therapy*	*1/2*	*50*
○ *Tebentafusp*	*1/2*	*50*
**Received at least three courses of systemic therapy**	**9/33**	**27.3**
– ICI as third systemic therapy	4/9	44.4
○ *Ipilimumab/Nivolumab*	*3/4*	*75*
○ *Nivolumab monotherapy*	*1/4*	*25*
– Chemotherapy as third systemic therapy	2/9	22.2
– Other therapies as third systemic therapy	3/9	33.3
○ *Tebentafusp*	*1/3*	*33.3*
○ *Nab-Paclitaxel and Alisertib*	*1/3*	*33.3*
○ *Niraparib*	*1/3*	*33.3*

Supplementary Table 2: Applied systemic therapies

Following abbreviation is used: Immune checkpoint inhibitors (ICI)
